# Selective Estradiol Sensitivity in 12Z Human Endometriotic Epithelial Cell Line

**DOI:** 10.3390/cells15201875

**Published:** 2026-10-15

**Authors:** Shohini Banerjee, Esha Chopra, Ian M. Smith, Kimberly M. Stroka

**Affiliations:** 1 Fischell Department of Bioengineering, University of Maryland, College Park, MD 20742, USA; shoban@umd.edu (S.B.); eshachopra2010@gmail.com (E.C.); smith.i@wustl.edu (I.M.S.); 2 Marlene and Stewart Greenebaum Comprehensive Cancer Center, University of Maryland, Baltimore, MD 21201, USA; 3 Biophysics Program, University of Maryland, College Park, MD 20742, USA; 4 Center for Stem Cell Biology and Regenerative Medicine, University of Maryland, Baltimore, MD 21201, USA

**Keywords:** estradiol, sensitivity, 12Z cell line, migration, confinement, estrogen receptor-alpha, endometriosis

## Abstract

**Highlights:**

**What are the main findings?**

- The 12Z cell line demonstrates restricted sensitivity to estradiol in most in vitro contexts, even after upregulation of the estrogen receptor gene.
- 12Z cells are selectively responsive to estradiol in confined migration models.

**What are the implications of the main findings?**

- The 12Z cell line, especially at high passages, should be used with caution when modeling estrogenic processes.
- Hormone sensitivity in 12Z cells needs to be verified in each experimental context.

**Abstract:**

Estradiol (E2) is a potent estrogen molecule that plays a crucial role in regulating numerous healthy and pathophysiological processes. To model estrogen-dependent cellular activities in vitro in a physiologically meaningful way, cells chosen for experimentation should exhibit sensitivity to E2. The 12Z human endometriotic epithelial cell line is gaining attention as a model for estrogen-dependent conditions, but its functional responsiveness to E2 has not been well characterized. For the passage range in the following study, we found that E2 does not statistically significantly influence 12Z proliferation, wound closure, or single-cell migration. However, 12Zs were selectively responsive to E2 in 3D migration models where the cells were physically confined. Upregulating *ESR1*, the primary mediator of estrogenic action, in the 12Zs did not enhance their functional E2 sensitivity. These results highlight the importance of verifying hormone responsiveness when using 12Z cells to model estrogen-dependent processes and conditions.

## 1. Introduction

Estrogens are key hormone molecules that are intimately involved in numerous healthy and disease physiological systems, including but not limited to the reproductive system, cardiovascular health, immune activity, cancer metastasis, endometriosis, polycystic ovary syndrome, and uterine fibroids [1,2,3,4]. Estradiol (E2), a potent estrogen, binds with estrogen receptors (ERs) located in the nucleus and on the plasma membrane to directly and indirectly regulate gene expression. This process impacts downstream cellular activities, such as cell proliferation, morphology, differentiation, migration, and invasion [5]. When modeling estradiol-dependent biological processes in vitro, it is important to use cells that retain their hormone sensitivity. While the advent of cell lines is advantageous for widespread use in high-throughput experiments, their physiological relevance can be limited by the loss of primary cell traits. The 12Z human endometriotic epithelial cell line has been developed as a model of endometriosis and estrogen-dependent metastasis and shares some molecular similarities with primary cells [6,7]. Although the cell line is gaining popularity, its responsiveness to E2 has not been well characterized. Given that endometriosis is marked by excessively high local levels of E2 that are known to impact primary cell behaviors [8,9,10,11,12,13], we sought to determine whether the 12Z cell line retains a similar sensitivity to E2. 12Z cells have previously been reported to express nuclear ERs [6,7], so we hypothesized that they would exhibit E2 dependence in vitro.

In the following study, we tested and characterized the E2 sensitivity of the 12Z cell line in a variety of proliferation and migration models. In our experimental context, we found that 12Zs exhibit selective responsiveness to E2, with altered behaviors in confined migration models but no changes seen in unconfined proliferation and migration systems. Moreover, increasing levels of the gene encoding ER⍺, a major mediator of estrogenic action [14], did not enhance 12Z responsiveness to E2. Lastly, we performed exploratory RNA sequencing. Given the limited responsiveness to E2, we question whether the 12Z cell line—at high passages—is appropriate for use in studying estrogen-dependent processes in vitro.

## 2. Materials and Methods

### 2.1. Cell Culture and Treatments

The 12Z cell line was purchased from Applied Biological Materials (Richmond, BC, Canada, Cat. no. T0764). Cells were cultured in DMEM/F-12 (Gibco, Waltham, MA, USA) supplemented with 10% fetal bovine serum (FBS) (Gibco) and 1% penicillin-streptomycin (Gibco). About 24–48 h prior to experiments, the media was replaced with phenol red-free DMEM/F-12 (Gibco) supplemented with 10% charcoal-stripped FBS (Gibco) and 1% penicillin-streptomycin (Gibco). Passages 55–75 were used for experiments. The 12Z cell line was authenticated (Laragen, Culver City, CA, USA) and tested mycoplasma-free. A modified 12Z cell line with an upregulation of *ESR1* (*ESR1*-12Z) was generously gifted by Dr. Fazleabas’s lab and cultured in phenol red-free DMEM/F-12 supplemented with 10% charcoal-stripped FBS and 1% penicillin-streptomycin. Passages 6–14 were used for experiments. The originating lab confirmed that cells were authenticated and mycoplasma-free prior to donation. After recovering from thawing, cell lines were cultured for approximately two weeks at a time, on average. 17β-estradiol (E2) was purchased from Sigma Aldrich (St. Louis, MO, USA, Cat. # E8875-1G) and dissolved in 100% ethanol to create a stock solution. Another vial of E2 was purchased from MP Biomedicals (Irvine, CA, USA, Cat. # 02194565-CF) and dissolved in 100% ethanol to create a stock solution (denoted as MPB E2). Cells were treated with media containing a vehicle (0.001% ethanol), 10 nM E2, or 10 nM MPB E2, as well as other treatments as specified in the Section 3 for the dose-dependent experiments. Unless specified as MPB E2, E2 refers to the batch sourced from Sigma Aldrich.

### 2.2. Proliferation Assay

To quantify cellular proliferation, a Cell Counting Kit-8 (Sigma Aldrich, Cat. # 96992) assay was employed. Briefly, 10,000 cells were seeded per well in a 96-well plate and cultured for 48 h with treatments. These include the mitotic inhibitor mitomycin C (MMC, Sigma Aldrich, cat. #M5353), which was used as a positive control at a concentration of 10 µg/mL. Then, 10 µL of CCK-8 solution was added to each well and incubated for 1–1.5 h at 37 °C. Absorbance measurements were taken with a microplate reader at 450 nm. Plotted absorbance values were normalized to the vehicle group, as specified in each figure.

### 2.3. Wound Closure Assay

Scratch wound healing assays were performed. Approximately 90,000 cells were seeded per well in a 24-well plate coated with 10 µg/mL collagen I from rat tail (Corning, NY, USA, cat. # 354249). Cells were incubated for 48 h with treatments as they grew into confluent monolayers. Monolayers were manually scratched with a p-200 pipette tip (VWR, Radnor, PA, USA) and rinsed with phosphate-buffered saline (PBS) before replacing media with treatments. Wounds were imaged under phase contrast at 10× one hour post-scratch (Initial wound area, t = 0) and 7.25 h post-scratch (Final wound area). Wound closure was calculated as (Initial wound area − Final wound area)/Initial wound area, with wound areas determined using ImageJ 2.16.0 (National Institutes of Health, Bethesda, MD, USA). Time-lapses were visually inspected and we did not observe extensive proliferation during the experiment.

### 2.4. Quantification of Cellular Morphology

Morphological parameters including cell area, perimeter, solidity, and roundness were extracted using ImageJ to manually trace cells from phase contrast microscope images. *Solidity* was defined as $\mathrm{solidity}=\;\frac{\mathrm{Cell}\;\mathrm{area}}{\mathrm{Convex}\;\mathrm{hull}\;\mathrm{area}}$, where a cell that is perfectly circular would have a value of 1.0 and a cell that has protrusions and/or indentations would have a value closer to 0. *Roundness* was defined as $\mathrm{roundness}=\;\frac{1}{\mathrm{Aspect}\;\mathrm{ratio}}=\;\frac{\mathrm{Minor}\;\mathrm{axis}}{\mathrm{Major}\;\mathrm{axis}}$, where a highly elongated cell would have a value close to 0.

### 2.5. Single-Cell Random Migration Analysis

The random migration of single cells was analyzed from time-lapse phase contrast images. Briefly, approximately 5000 cells were seeded per well in a 24-well plate coated with 10 µg/mL rat tail collagen I, except for the positive control, where the wells were coated with 20 µg/mL poly-D lysine (PDL) to prevent integrin-based cell attachment. Cells were incubated with treatments for 48 h and then imaged under phase contrast at 10× for 12 h, with a time step of 10 min. Individual cell trajectories were tracked using the Manual Tracking plugin in ImageJ by clicking on the estimated geometric center of the cell at each time point. Subsequent analysis was performed in MATLAB (R2024a). Average speed was defined as the cell’s total path length divided by the duration of the time-lapse. Data represent speed normalized to the vehicle. Directional persistence was defined as the cell’s end-to-end displacement divided by its total path length, where a cell that progresses in a perfectly straight line from point A to point B would have a directional persistence of 1; a cell that originates at a point A, moves in a straight line to point B, and directly returns to point A would have a directional persistence of 0. Mean-square displacement (MSD), which is a proxy for how much area (in µm^2^) a cell covered during its trajectory, is defined as *MSD* = 〈*r*^2^〉 = 4*D*Δ*t*, where Δ*t* is the number of time steps (Δ*t* = 1 represents one step, or 10 min; Δ*t* = 2 represents two time steps, or 20 min, etc.) and *D* represents the diffusivity (µm^2^/time step). Diffusivity was derived from the slope *m* of the linear portion of the MSD curve, where *m* = 4*D* and $D=\frac{\langle r^{2}\rangle }{4∆t}$.

### 2.6. Transwell Migration Assay

Cell transmigration through a semi-permeable membrane was performed using a transwell migration assay. Transwell inserts for a 24-well plate were coated with 10 µg/mL collagen I from rat tail. 12Z cells were treated with vehicle or E2 for 48 h prior to placement in the transwells. Then, 100,000 cells were seeded in the upper chamber in serum-free media containing treatments, with the lower chamber containing media supplemented with 10% FBS. Cells were allowed to transmigrate for 24 h prior to quantification. Then, media was aspirated from the upper chambers, the tops of the transwell inserts were swabbed gently, and the inserts were stained with 5 µM calcein AM for 30 min. Transmigrated cells were imaged under fluorescence. Data were normalized to the vehicle.

### 2.7. Cell Migration Through Confined Microfluidic Channels and Analysis

To analyze 12Z migration in narrow spaces, cells were sent through microfluidic devices containing narrow channels. As previously defined [15], microfluidic devices with confining microchannels 3 µm wide × 10 µm tall × 200 µm long were cast in polydimethylsiloxane (PDMS, Dow) using a positive mold created by photolithography. The inlets and outlets of the PDMS device were made with a 4 mm biopsy punch. The devices were then irreversibly adhered to a #1 glass coverslip via plasma treatment, followed by 5 min of pressure and an overnight bake at 80 °C. One hour before their use, the devices were again plasma-treated to create hydrophilic surfaces, and 0.1 mg/mL type I collagen (cat. # C3867, Sigma Aldrich) in PBS was added to the inlets to functionalize the inner walls. Devices with collagen were incubated for 1 h at room temperature. The devices were then added to a 4-well plate (07-000-207, Fisher Scientific, Waltham, MA, USA) and UV-treated for 10 min to sterilize them before adding cells. Following initial cell splitting, cells were resuspended in serum-free media, counted, and centrifuged again. Cells were then resuspended to a concentration of 100,000 cells/20 µL of serum-free media containing vehicle or E2 treatments. Then, 20 µL of this cell suspension was added to the microchannel cell inlet. After adding cells, the devices were placed in an incubator for 10 min to ensure cell adhesion to the microchannel walls. Afterwards, excess media and adhered cells were removed, and media was added to the remaining wells. Cell inlets and outlets, in addition to 2 media inlets and the media outlet, were filled with serum-free media. The final media inlet was filled with full media to induce a chemokine gradient. Approximately ten regions of interest were selected for each device and imaged at 20× under phase contrast every 15 min for 16 h.

Individual cell trajectories while in the microchannels were tracked using the Manual Tracking PlugIn in ImageJ by clicking on the nucleus of the cell at each time point. Subsequent analysis was performed in MATLAB. Speed was defined as the cell’s total path length divided by the duration of the time-lapse. Data represent speed normalized to the vehicle. Directional persistence was defined as the cell’s end-to-end displacement divided by its total path length, where a cell that progresses in a perfectly straight line from point A to point B would have a directional persistence of 1; a cell that originates at a point A, moves in a straight line to point B, and directly returns to point A would have a directional persistence of 0.

### 2.8. Spheroid Outgrowth Assay

A spheroid outgrowth experiment was performed to quantify the expansion of 3D cell spheroids on a collagen I gel. Briefly, 4000 12Z cells were seeded per well in a round-bottom 96-well ultra-low attachment plate and centrifuged at 150× *g* for 2 min. Spheroids were harvested at 48 h and seeded on top of collagen I gels. Phase contrast images were taken at time of seeding (Day 0) and at 24 h (Day 1). The projected area of each spheroid was measured in ImageJ; data represent the area on Day 1 relative to the initial area on Day 0.

### 2.9. Western Blotting

Relative levels of estrogen receptor-α (ERα) were measured with Western blotting to validate the overexpression of its encoding gene, *ESR1*, in the *ESR1*-12Zs. Cells were cultured for 48 h and lysed with a protease cocktail inhibitor (1:100; Sigma Aldrich) and phenylmethylsulphonyl fluoride (1:500; Sigma Aldrich, cat. #P7626) in Pierce RIPA buffer (ThermoFisher Scientific, Waltham, MA, USA). A BCA Protein Assay Kit (ThermoFisher Scientific) was used to determine protein concentration in the lysates. Samples were mixed 1:1 with a buffer containing 20:1 2× Laemmli to β-mercaptoethanol (Bio-Rad, Hercules, CA, USA, cat. #1610737 and #1610710, respectively) and boiled. Equal amounts of protein were loaded into a Mini-PROTEAN TGX 4-20% precast gel (Bio-Rad) and separated by sodium dodecyl sulfate-polyacrylamide gel electrophoresis. Samples were then transferred to a PVDF membrane via the Trans-Blot Turbo machine (Bio-Rad) on the Mixed MW Turbo protocol setting (1.3 A, up to 25 V, 7 min). PVDF membranes were blocked in an Intercept (PBS) blocking buffer (LI-COR, Lincoln, NE, USA) for one hour and incubated overnight at 4 °C with a primary antibody solution consisting of anti-ERα (1:500; Santa Cruz Biotechnology, Dallas, TX, USA; cat. #sc-8002) and anti-α-smooth muscle actin (1:500; Sigma Aldrich, cat. #A5228) as a control in a 1:1 Intercept blocking buffer to 0.1% Tween 20 in PBS mix (PBST). The next day, membranes were washed with PBST and incubated for one hour at RT with a secondary antibody solution consisting of IRDye 680RD Goat anti-Rabbit and Goat anti-Mouse (each at 1:10,000; LI-COR, cat. #926-68071 and #926-68070, respectively) in a 1:1 Intercept blocking buffer to PBST mix. Membranes were then washed with PBS, visualized on an Odyssey CLx imager (LI-COR), and analyzed with the Image Studio 5.0 software (LI-COR).

### 2.10. RNA-Sequencing and Data Analysis

Vehicle- and E2-treated 12Zs, as well as non-treated 12Zs and non-treated *ESR1*-12Z samples, were sent for RNA extraction, integrity analysis, library preparation, and sequencing (Novogene Co., Ltd., Sacramento, CA, USA). Three independent samples for each cell group were pooled before shipping. Messenger RNA was purified from total RNA using poly-T oligo-attached magnetic beads. After fragmentation, the first-strand cDNA was synthesized using random hexamer primers. Then, the second-strand cDNA was synthesized using dUTP instead of dTTP. The directional library was ready after end repair, A-tailing, adapter ligation, size selection, USER enzyme digestion, amplification, and purification. The library was checked with Qubit and real-time PCR for quantification and bioanalyzer for size distribution detection. After library quality control, libraries were subjected to Illumina Novoseq 6000 sequencing (paired-end 150 bp) with a data output of over 20 million read pairs per sample. Raw reads (fastq format) were processed through fastp software. Clean reads were obtained by removing reads containing adapter, reads containing ploy-N and low-quality reads from raw data.

RNA from three independently prepared cell samples was pooled prior to sequencing, resulting in one pooled sequencing library per condition. Thus, the three samples comprising each pool were not retained as independent biological replicates for transcriptomic analysis. Gene expression analysis was performed by Novogene using edgeR (v4.0.16), with read counts adjusted using scaling normalization factors to account for differences in sequencing depth. Genes meeting the provider’s thresholds of |log2(fold change)| ≥ 1 were identified. Because only one pooled library was sequenced per condition, biological variability and condition-specific dispersion could not be independently estimated from biological replicates. Accordingly, these analyses are presented as exploratory transcriptomic comparisons rather than confirmatory differential-expression analyses. Full RNA sequencing methods are provided in the Supplementary Material S1.

### 2.11. Microscopy

For all phase contrast images and transmigration fluorescence images, live cells were imaged using the 10× objective (unless otherwise specified) on an Olympus IX-83 inverted microscope (Olympus, Center Valley, PA, USA) and the Olympus cellSens 4.5 software. Cells were maintained at 37 °C, 65% humidity, and 5% CO_2_ in an enclosed chamber surrounding the microscope.

### 2.12. Statistical Analysis

GraphPad Prism 10 (La Jolla, CA, USA) was utilized to generate graphs and perform statistical analyses on biological replicates. Outliers were identified using the ROUT method with a Q (maximum desired false discovery rate) = 1% only to remove definite outliers. Data were tested for normality. For comparisons between vehicle and E2 groups, unpaired Student’s *t*-tests were performed. For comparisons between three or more groups, ordinary one-way ANOVA with Tukey’s multiple comparisons test was performed. Corresponding non-parametric tests were used for non-normally distributed datasets. For all tests, *p* values < 0.05 were considered to be statistically significant. Data are represented as mean ± standard error of the mean. All experiments were performed using three independent biological replicates. Each biological replicate consisted of three technical replicates (different wells in a well plate).

## 3. Results

### 3.1. Determination of E2 Incubation Time and Concentration for 12Zs

**Figure 1:**
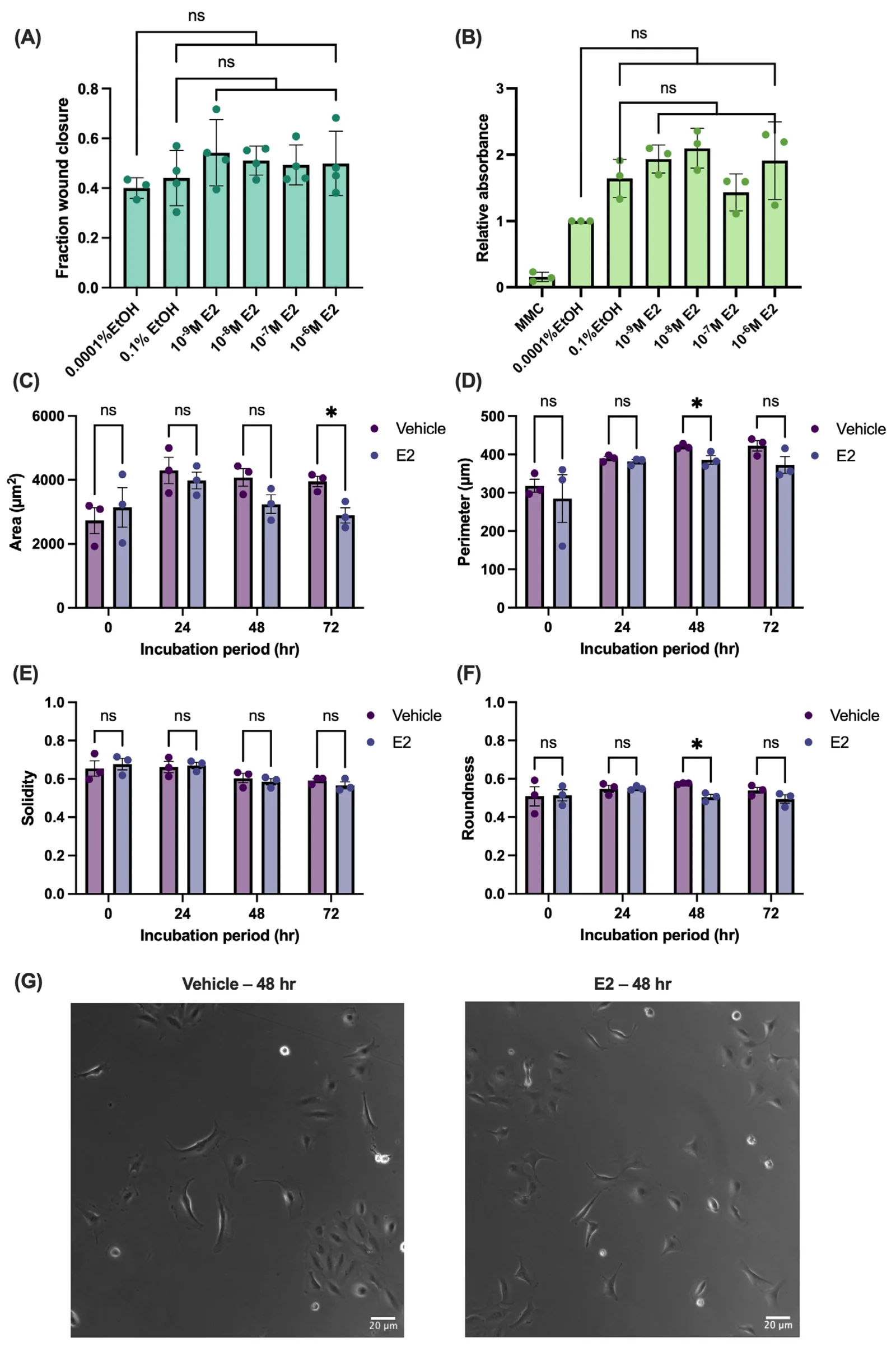
12Z characterization and determination of E2 dose and incubation time. (**A**) A wound healing assay and (**B**) absorbance from a CCK-8 assay, reported relative to the 0.0001% EtOH group (with mitomycin C as a positive control), were quantified. From this point on, all subsequent experiments were performed using a concentration of 10 nM E2 and a corresponding 0.001% EtOH vehicle control. We then imaged cells treated with this vehicle control and 10 nM E2 for multiple incubation times and quantified cell morphology parameters including (**C**) area, (**D**) perimeter, (**E**) solidity, and (**F**) roundness. (**G**) Representative images from the 48 h incubation period are shown. From this point on, 48 h was selected as the treatment incubation period for all subsequent experiments. Scalebars = 20 µm. Data points represent biological replicates (*n* = 3); error bars represent standard error of the mean. Ordinary one-way ANOVA with Tukey’s multiple comparisons test was performed for data in panels (**A**,**B**). For the rest of the data, an unpaired Student’s *t*-test was performed. * *p* ≤ 0.05; ns, not significant. EtOH; ethanol. E2; estradiol. MMC; mitomycin C.

Multiple functional assays were employed to determine the optimal incubation time and concentration of E2 to apply to 12Z cells. To determine the most effective dose of E2, cells were treated with 10^−9^ M, 10^−8^ M, 10^−7^ M, or 10^−6^ M E2 for 48 h before testing their migratory and proliferative response. Instead of including all the corresponding vehicle controls, only the lower and upper extremes were tested (0.0001% EtOH corresponding to 10^−9^ M E2 and 0.1% EtOH corresponding to 10^−6^ M E2). Differences in wound closure and proliferation between the 0.0001% EtOH and 0.1% EtOH vehicle groups were not significant. None of the E2 concentrations significantly impacted wound closure compared to either of the vehicle controls (Figure 1A). In a CCK-8 proliferation assay, 10^−9^ M and 10^−8^ M E2 treatments each led to a non-significant increase in absorbance compared to the 0.0001% and 0.1% EtOH control groups (Figure 1B). Based primarily on concentrations used in the literature [9,10,11,13,16,17], we selected 10^−8^ M (10 nM) as our concentration for all subsequent experiments involving E2. To determine the most effective incubation time with chosen treatments (0.001% EtOH vehicle control and 10 nM E2) for the 12Z cells, we analyzed cell morphology at the time of adding treatments (t = 0), 24 h, 48 h, and 72 h from phase contrast images. Results show that E2 induces slight changes in cell morphology after 48–72 h (Figure 1C–G). Cell area (Figure 1C) and perimeter (Figure 1D) decreased at 72 and 48 h, respectively. There were no changes in solidity across the incubation times (Figure 1E) but a decrease in roundness at 48 h (Figure 1F). Representative images of 12Z cells treated for 48 h are shown (Figure 1G). Given the decrease in cell perimeter and roundness at 48 h, along with support from the literature [10,13,17,18], we chose 48 h as the incubation time for all subsequent experiments involving E2.

### 3.2. E2 Has No Statistically Significant Effect on 12Z Proliferation, Collective Wound Closure, or Single-Cell Random Migration

**Figure 2:**
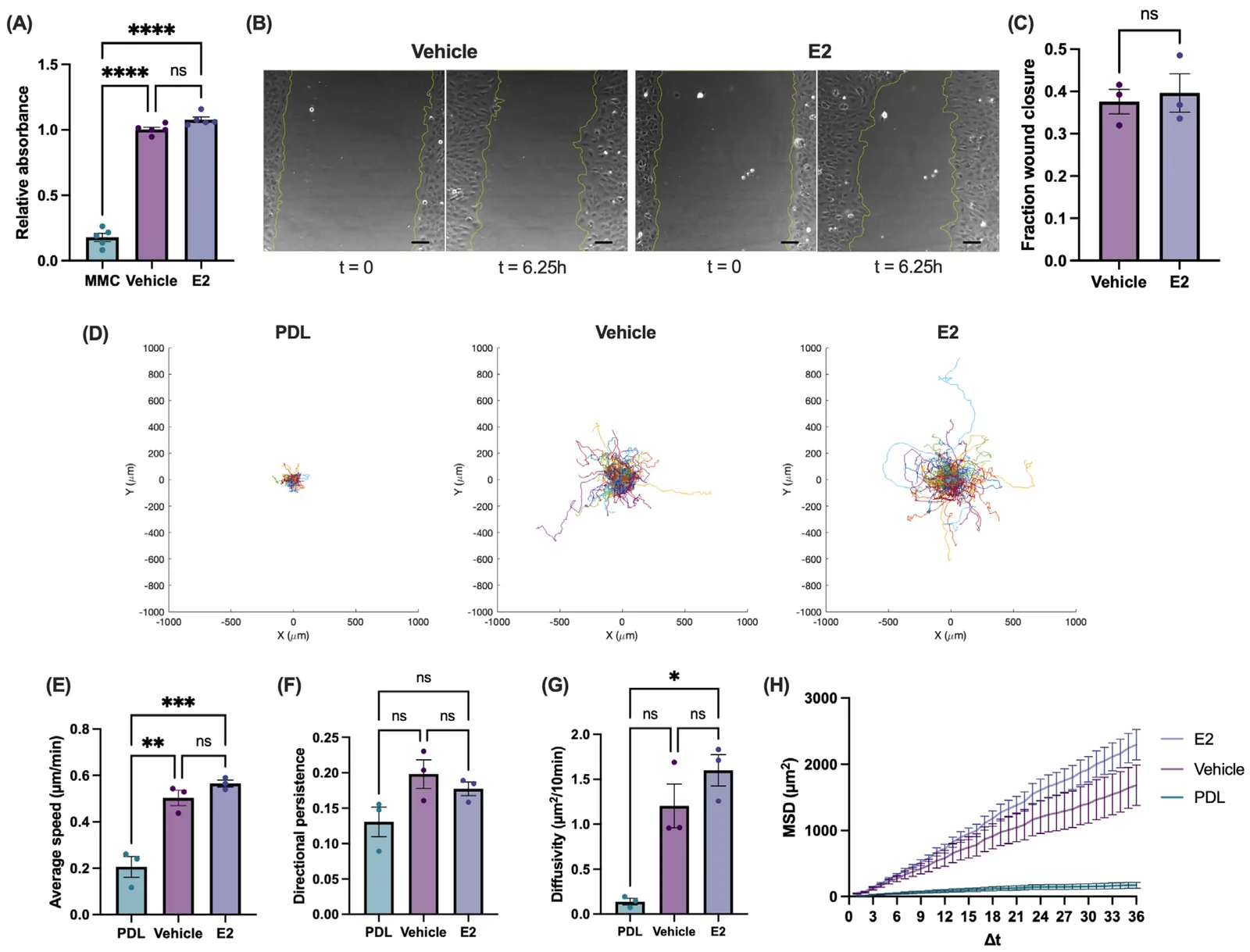
Effect of E2 on proliferative and migratory behaviors of 12Zs. 12Z cells were treated with 10 nM E2 for 48 h and their functional behaviors were assessed. (**A**) Proliferation was measured with a CCK-8 assay (with 10 µg/mL MMC as a positive control), and absorbance values are plotted relative to the vehicle control group. (**B**) Representative pictures of wound closure are shown from one hour post-scratch at t = 0 and midway through the experiment at t = 6.25 h for both vehicle and E2-treated 12Zs. (**C**) Fraction wound closure is calculated. (**D**–**H**) Random migration was measured via single-cell tracking, and a poly-D lysine coating, which prevents integrin-based attachment, was used as the positive control. (**D**) Single-cell trajectories are shown. (**E**) Speed, (**F**) directional persistence (defined as the cell’s total displacement divided by the cell’s total path length), (**G**) diffusivity, and (**H**) MSD over increasing time steps are plotted. Scalebars = 100 µm. Data points represent biological replicates (*n* = 3); error bars represent standard error of the mean. Ordinary one-way ANOVA with Tukey’s multiple comparisons test was performed for data in panels (**A**,**E**,**F**). For data in panel (**C**), an unpaired Student’s *t*-test was performed. For data in panel (**G**), a Kruskal–Wallis test with a Dunn’s multiple comparisons test was performed. * *p* ≤ 0.05, ** *p* ≤ 0.01, *** *p* ≤ 0.001, **** *p* ≤ 0.0001, ns, not significant. MMC; mitomycin C. E2; estradiol. PDL; poly-D lysine.

The responsiveness of 12Zs to 10 nM E2 treatment for 48 h was assessed in vitro with proliferation and migration assays. E2 had no statistically significant influence on 12Z proliferation in a CCK-8 assay, in which MMC was used as a positive control and absorbance values were plotted relative to the vehicle control group (Figure 2A). E2 also had no significant impact on wound closure (Figure 2B,C). We then assessed single-cell random migration in response to E2 (Figure 2D–H) and used a poly-D lysine (PDL) coating to prevent integrin-based cell–substrate attachment as a positive control. Individual cell trajectories show that E2-treated cells covered slightly more area than vehicle treated cells (Figure 2D). E2 did not significantly impact cell speed (Figure 2E), directional persistence (Figure 2F), or diffusivity (Figure 2G) compared to the vehicle control group. The mean-square displacement (MSD) graph shows that the E2-treated cells traversed the largest area, but not significantly more than the vehicle-treated cells (Figure 2H). The 12Z cell line did not appear to be sensitive to E2 treatment in proliferation and 2D migration assays in vitro. In case our E2 sourced from Sigma Aldrich was defective, we decided to additionally test an alternative source of E2 purchased from MP Biomedicals, denoted as MPB E2. However, MPB E2 had no significant effect on 12Z wound closure (Supplementary Material S2A).

### 3.3. E2 Differentially Affects 12Z Migration in Confined and Unconfined Systems

**Figure 3:**
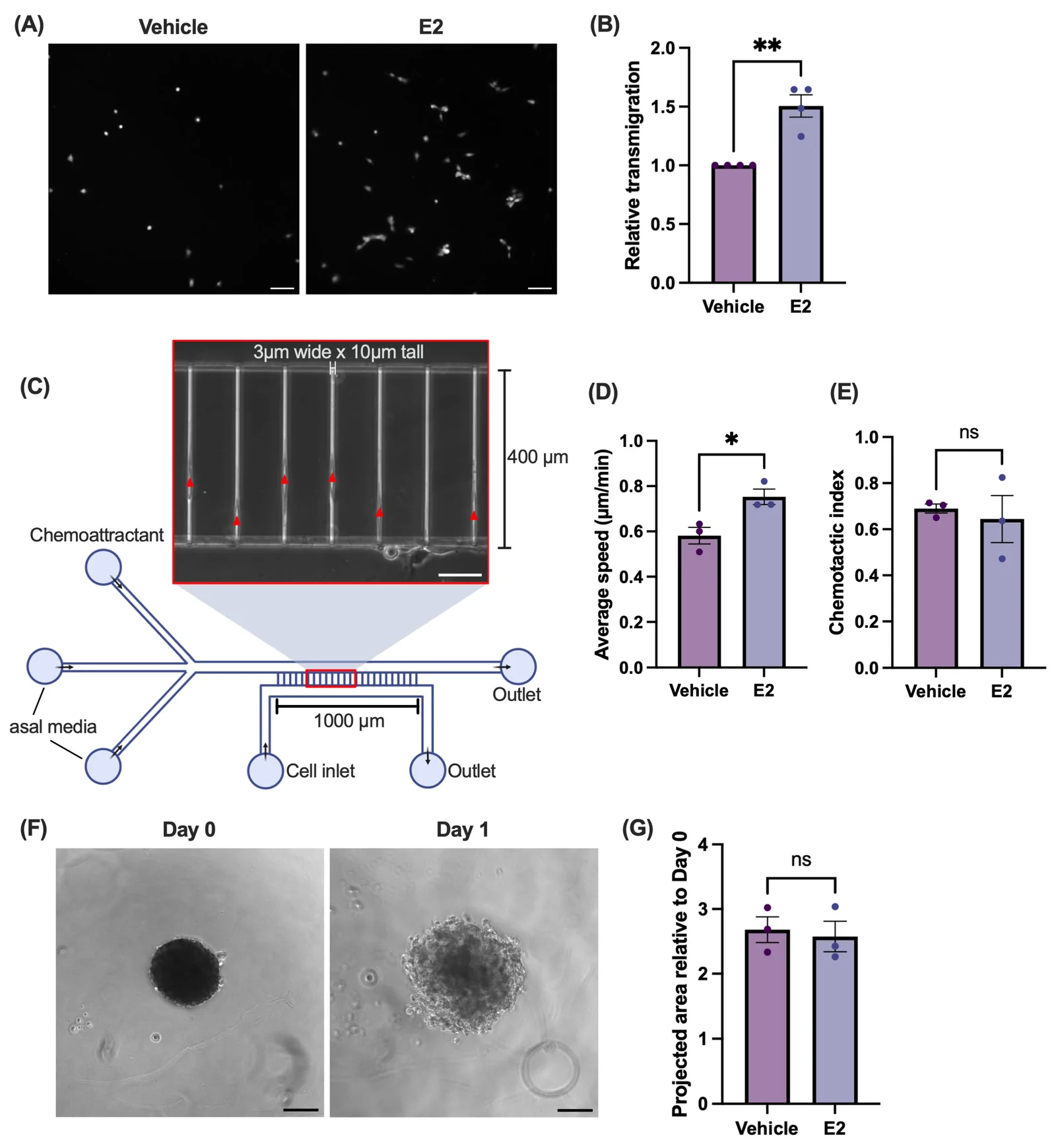
Impact of E2 on 12Zs in confined and unconfined systems. 12Z cells were treated with the vehicle or E2 for 48 h prior to experiments. (**A**) Transmigration in a transwell assay was measured and (**B**) quantified relative to the vehicle control. (**C**–**E**) Spontaneous migration of 12Zs through confining microchannels was observed. (**C**) The design of the microfluidic device is depicted in the schematic which contains a representative image of cells moving through the microchannels towards the chemoattractant (media with 10% charcoal-stripped FBS); the cell nuclei are marked by red triangles. (**D**) Cell speed and (**E**) chemotactic index, defined as the end-to-end displacement divided by the total cell path length, were quantified. (**F**) 3D 12Z spheroids were grown and seeded on collagen I gels (Day 0) and their projected area of outgrowth was measured the next day (Day 1). (**G**) Projected area is calculated relative to Day 0. Scalebars = 100 µm in panels (**A**,**F**) and 50 µm in panel (**C**). Data points represent biological replicates (*n* = 3); error bars represent standard error of the mean. Unpaired Student’s *t*-tests were performed on the data. * *p* ≤ 0.05, ** *p* ≤ 0.01, ns, not significant. E2; estradiol.

The 12Z cell line showed a lack of sensitivity to E2 in proliferation and 2D migration assays, but cells are known to behave in a context-dependent manner; this could include differences between confined and unconfined systems or between 2D and 3D [19,20,21,22]. After treating 12Zs with 10 nM E2 for 48 h, their migration in confined and unconfined systems was analyzed (Figure 3). E2 significantly increased transmigration in a transwell assay, with representative images showing the bottom-facing surfaces of the 8 µm-pore transwell inserts (Figure 3A). Transmigration was quantified relative to the vehicle control (Figure 3B). The effect of E2 was consistent when we tested the alternative source of E2, MPB E2, on 12Z transmigration (Supplementary Material S2B). In another confined migration model, we seeded 12Z cells in a previously defined PDMS microfluidic device containing narrow microchannels, 3 µm wide by 10 µm tall (Figure 3C) [15]. E2 increased cell migration speed through the channels (Figure 3D) but did not impact chemotactic index, defined as the end-to-end displacement divided by the total cell path length (Figure 3E). To model unconfined 3D migration, we seeded 12Z spheroids onto collagen I gels (Day 0) and measured their outgrowth 24 h later (Day 1) (Figure 3F). E2-treated spheroids did not exhibit differences in outgrowth compared to vehicle-treated spheroids (Figure 3G).

### 3.4. Upregulation of ESR1 Does Not Induce Functional E2 Sensitivity in the 12Zs

**Figure 4:**
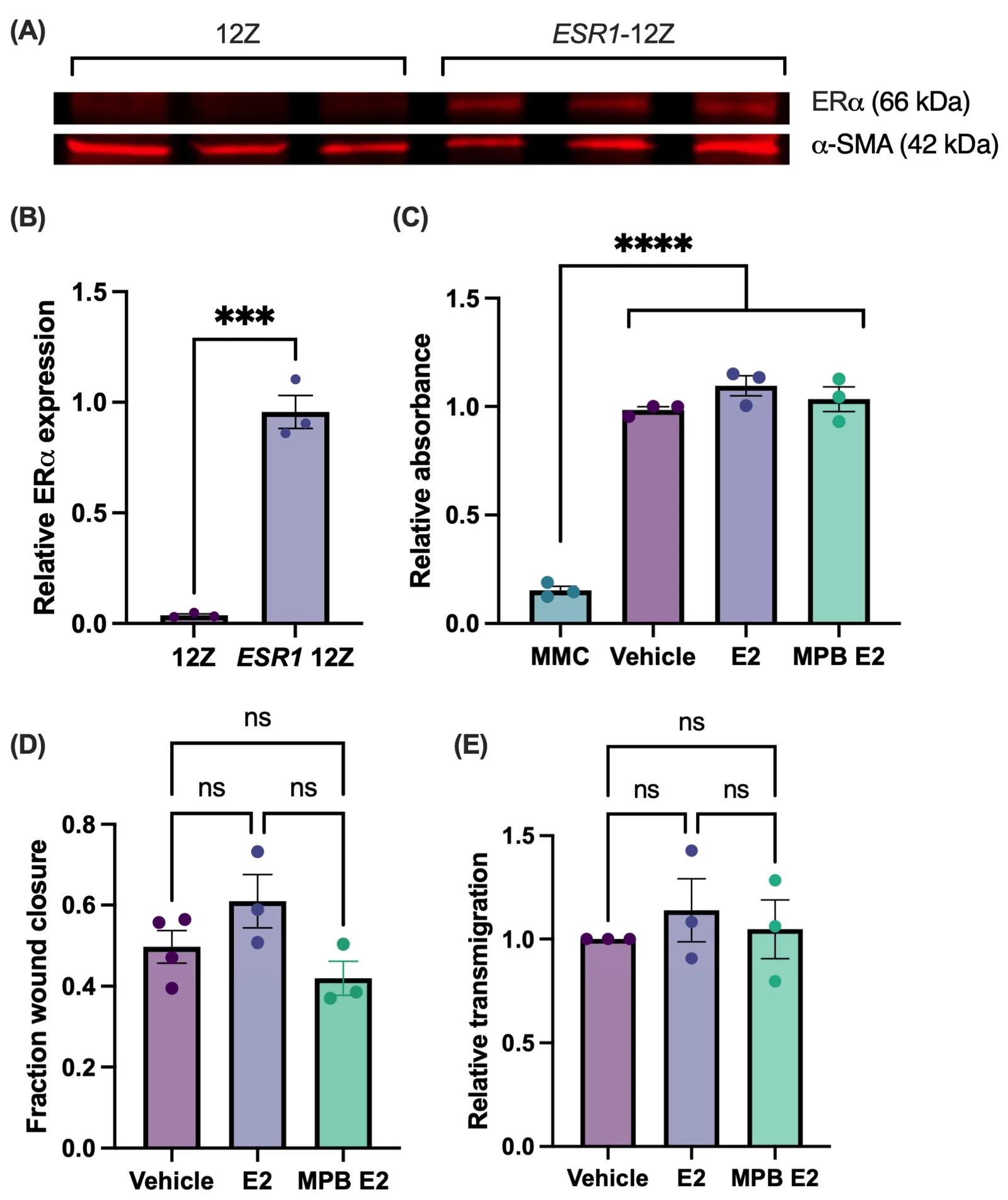
Effect of E2 on proliferative and migratory behaviors of *ESR1*-12Zs. We acquired 12Z cells stably transfected with *ESR1* (*ESR1*-12Zs) [23], the gene encoding the estrogen receptor ⍺ (ER⍺). (**A**) A Western blot for ER⍺ protein expression was performed using ⍺-smooth muscle actin (⍺-SMA) as the reference protein and (**B**) quantified relative to ⍺-SMA in the *ESR1*-12Zs. (**C**–**E**) Then, *ESR1*-12Zs were subject to vehicle, 10 nM E2, or 10 nM MPB E2 for 48 h and tested for (**C**) proliferation in a CCK-8 assay (with 10 µg/mL MMC as a positive control) reported relative to the vehicle control, (**D**) wound closure, and (**E**) transwell migration reported relative to the vehicle control. Data points represent biological replicates (*n* = 3); error bars represent standard error of the mean. For panel (**A**), an unpaired Student’s *t*-test was performed. Ordinary one-way ANOVA with Tukey’s multiple comparisons test was performed for data in panels (**C**–**E**). *** *p* ≤ 0.001, **** *p* ≤ 0.0001, ns, not significant. E2; estradiol (original batch purchased from Sigma Aldrich). MPB E2; MP Biomedicals estradiol. MMC; mitomycin C.

Our results show that the 12Z cell line is unresponsive to E2 treatment in most standard in vitro models of proliferation and unconfined migration. Estrogenic action is thought to be predominantly mediated by *ESR1*, the gene that encodes ER⍺ [14]. We hypothesized that increasing the levels of ER⍺ would enhance the responsiveness of 12Zs to E2. We obtained a 12Z cell line genetically modified to upregulate *ESR1* [23] from Dr. Fazleabas’s lab (Michigan State University), hereafter denoted as *ESR1*-12Z. Upon receiving the *ESR1*-12Z cell line, we verified the increased protein expression of ER⍺ with a Western blot (Figure 4A). Western blot replicates and the effect of E2 on ER⍺ protein expression are reported (Supplementary Material S3). The *ESR1*-12Zs significantly overexpress ER⍺ protein compared to the normal 12Zs; in fact, ER⍺ appears to be nearly absent in the 12Zs (Figure 4B). However, after treating the *ESR1*-12Zs with 10 nM E2 (an alternate source of E2, denoted MPB E2, was also tested) for 48 h, their functional behaviors showed no changes (Figure 4C–E) compared to the vehicle control. In a CCK-8 proliferation assay with MMC as a positive control, neither source of E2 led to a change in absorbance compared to the vehicle control (Figure 4C). Similarly, neither source of E2 significantly impacted wound closure (Figure 4D) or migration through a transwell insert (Figure 4E).

### 3.5. Gene Expression Profiles: Effects of E2 Treatment or ESR1 Upregulation

**Figure 5:**
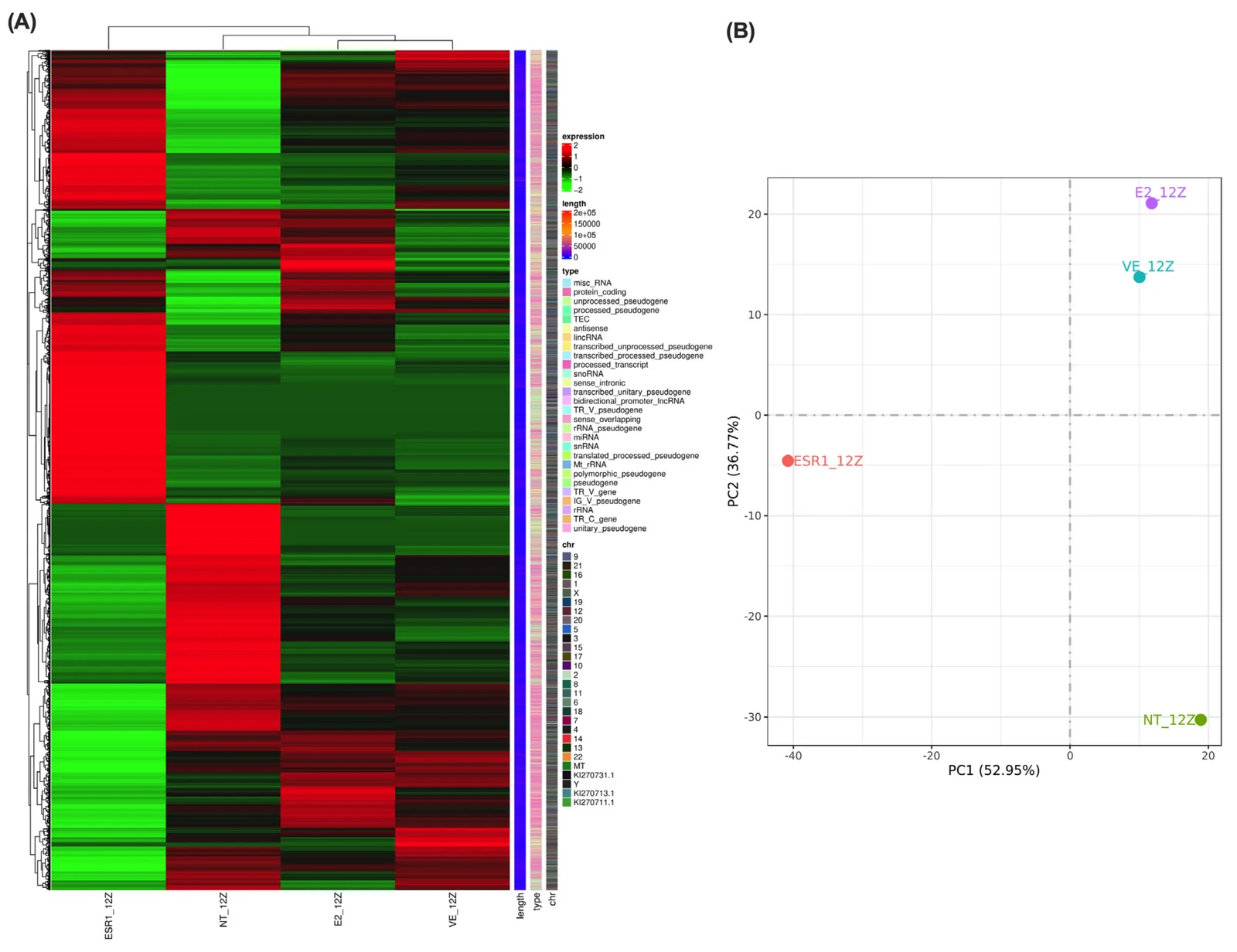
Gene expression profiles of vehicle-treated 12Zs, E2-treated 12Zs, normal 12Zs, and *ESR1*-12Zs. Vehicle-treated 12Zs (VE_12Z), E2-treated 12Zs (E2_12Z), normal 12Zs (NT_12Z), and *ESR1*-12Zs (ESR1_12Z) were sent for RNA extraction and sequencing (Novogene Co., Ltd.). (**A**) Gene expression profiles can be visualized in the heatmap of mainstream hierarchical clustering and (**B**) intergroup differences can be seen in the 2D principal component analysis plot. Each cell sample contains 3 trials pooled prior to sequencing.

Vehicle-treated 12Zs (VE_12Z), E2-treated 12Zs (E2_12Z), normal 12Zs (NT_12Z), and *ESR1*-12Zs (ESR1_12Z) were sent for RNA extraction and sequencing (Novogene Co., Ltd.). Three biological replicates were pooled, resulting in one pooled sequencing library per condition. Therefore, the RNA sequencing was exploratory and descriptive rather than mechanistic. Mainstream hierarchical clustering analysis shows some transcriptomic similarity between VE_12Z and E2_12Z (11,512 genes co-expressed, 451 and 580 genes uniquely expressed, respectively) and comparatively less similarity between NT_12Z and ESR1-12Z (10,818 genes co-expressed, 1034 and 1065 genes uniquely expressed, respectively) (Figure 5A). These intergroup differences are also shown in the 2D principal component analysis (Figure 5B).

A gene expression analysis was carried out using the EdgeR trimmed mean of M values method [24] to normalize the data. In the E2_12Z group compared to the VE_12Z group, there were 289 genes with a |log2FoldChange| > 1: 185 upregulated and 104 downregulated (Supplementary Material S4). Out of the top 10 genes with a |log2FoldChange| > 1, five were non-coding RNA or pseudogenes such as *AC010331.1*, *AL391280.1*, and *AC008555.8*, which were all upregulated in E2_12Z. The roles of these non-coding RNAs are not well defined, although they are often capable of indirectly regulating gene expression [25,26]. Other genes in the top 10 with a |log2FoldChange| > 1 were protein-coding genes involved in metabolism and cellular signaling. Examples include *N4BP3*, *PDE4C*, and *GATM*, which were upregulated in E2_12Z. However, further validation is needed to provide mechanistic insight.

The gene expression analysis between NT_12Z and ESR1_12Z revealed much larger transcriptomic differences. There were 6779 genes with a |log2FoldChange| > 1: 3229 upregulated and 3550 downregulated (Supplementary Material S5). *PLCB1*, *ANXA10*, *WISP1*, and *HAS2* were particularly downregulated in ESR1_12Z compared to NT_12Z and are involved in signal transduction, cell migration, and cell invasion [27,28,29,30]. Notable upregulated genes include *TMPRSS15*, a protease that activates other proteases and cleaves proteins involved in cell signaling [31], *EPHA3*, which inhibits cell migration [32], and *ESR1*. To further characterize the genes with large fold changes, an exploratory KEGG (Kyoto Encyclopedia of Genes and Genomes) [33] analysis was conducted. Out of the 14 pathways that were enriched, several involved cellular signaling and cell–substrate adhesion functions such as cytokine–cytokine receptor interaction, focal adhesion, cell adhesion molecules, and ECM–receptor interaction. All of the enriched KEGG pathways are provided for this comparison (Supplementary Material S6). Further validation is required to attribute these transcriptomic differences solely to the upregulation of *ESR1* in the 12Zs.

## 4. Discussion

Estradiol (E2), a potent estrogen molecule, plays a fundamental role in regulating diverse physiological processes and contributes to the development of benign and malignant conditions. Despite its broad impact, the precise mechanisms by which E2 modulates cellular activities in different tissue contexts remain incompletely understood. As researchers increasingly turn to in vitro models to study estrogen-dependent processes, it is critical to ensure that the cell lines used retain physiologically relevant responsiveness to E2. Without this validation, conclusions cannot be drawn about estrogenic effects.

In the present study, we sought to characterize the sensitivity of the 12Z human endometriotic epithelial cell line to E2. While this cell line is frequently used in the study of estrogen-mediated conditions like endometriosis, our results challenge the extent of its assumed responsiveness to E2 in common in vitro models. We observed no significant changes in proliferation, wound closure, or single-cell random migration upon treatment with 10 nM E2 for 48 h (Figure 2). Together, these data suggest that the 12Z cell line exhibits limited sensitivity to E2 in standard 2D in vitro systems, contrary to the sensitivity seen in primary endometriotic epithelial cells [13,17,34,35,36]. A previous study demonstrated that E2 treatment of 12Zs did not enhance their production of pro-inflammatory cytokines [37]. While this is a different experimental system, it reflects a similar theme of restricted responsiveness to E2 in the 12Z cell line. Another study found that E2 treatment increased the invasion of heterogeneous spheroids composed of 12Z cells and endometriotic stromal cells within Matrigel containing peritoneal mesothelial cells [38]. However, it is unclear whether the 12Zs, the stromal cells, or both were hormone responsive. In fact, the stromal cells might have been more sensitive to E2-induced invasiveness, considering that they were localized to the leading edges of the spheroids [38]. Although 10 nM E2 and a 48 h treatment period were selected for this study based on preliminary dose- and time-response experiments and the previous literature, these experiments do not exclude the possibility that other E2 concentrations, exposure durations, or temporal patterns of stimulation could elicit functional responses not captured under the conditions examined here.

Interestingly, our results showed that the 12Zs did demonstrate a selective E2 responsiveness in physically confined migration systems (Figure 3). This suggests that E2 may modulate cell behavior in a context-dependent manner, particularly in environments that mimic mechanical constraints present in vivo. The mechanism underlying this context-dependent response remains unknown. Mechanical confinement could potentially alter mechanotransduction or estrogen-associated signaling; however, these pathways were not directly investigated in the present study and require future molecular validation. This phenomenon underscores the importance of considering physical context when evaluating hormone responsiveness in vitro, particularly given that 12Z cell migration itself is sensitive to mechanical properties of the extracellular environment [39]. Moreover, others have shown a differential expression of estrogen-related genes in 3D compared to 2D settings [22]. Exploratory RNA sequencing revealed greater expression of pseudogenes or non-coding RNAs in E2-treated 12Z cells. While their exact functions remain unclear, these molecules are increasingly recognized as regulators of gene expression through competing endogenous RNA activity, epigenetic modulation, or chromatin remodeling [25,40]. Whether these transcriptomic observations relate to the context-dependent functional response to E2 remains unknown and warrants further investigation. These observations differ from a previous study that reported an upregulation of some genes related to cell proliferation, apoptosis, motility, and signaling upon E2 treatment of the 12Zs [41]. These contrasting results could be due to the use of different methods (proteomic analysis with mass spectrometry versus transcriptomic analysis with RNA sequencing in this study), different treatment incubation times (24 vs. 48 h), pooling differences, or different passage numbers.

Although the overexpression of *ESR1* substantially increased ER⍺ protein levels, it did not translate to increased functional responsiveness to E2 in proliferation or migration assays (Figure 4). We hypothesize that restricted E2 sensitivity in the 12Zs is not solely due to low or absent ER⍺ levels but could reflect downstream desensitization of estrogen signaling pathways. Exploratory RNA-sequencing analysis identified differences in several genes involved in signal transduction and membrane trafficking, including *PLCB1* and *ANXA10*. Enriched KEGG pathways involved cytokine interactions, focal adhesions, cell adhesion molecules, and ECM-receptor signaling. These observations generate candidate pathways that could be investigated in future studies using independently replicated transcriptomic and targeted molecular analyses. Given the pooled RNA-seq design, however, these expression differences cannot establish a mechanism underlying the observed functional phenotype. Given the discrepancy between *ESR1* expression and the lack of functional response to E2, future studies should investigate (1) the crosstalk between ER⍺, ERβ, and G protein-coupled estrogen receptor expression and how they determine cellular E2 sensitivity and (2) the balance between classical (genomic) and non-classical (non-genomic) ER signaling pathways in the 12Zs. Classical ER signaling involves the direct binding of ligand-bound ERs to DNA at estrogen response elements (EREs), while non-classical signaling typically occurs through membrane-bound ERs that activate kinase cascades and secondary messengers [1]. Investigating these signaling pathways under confined and unconfined conditions could help determine whether they contribute to the context-dependent E2 responsiveness observed here.

Overall, these findings highlight the importance of validating E2 responsiveness when using the 12Z cell line as a model for estrogen-dependent processes. While it may still serve as a tool in endometriosis research, its limited E2 responsiveness in most in vitro contexts raises concerns about its utility for studying canonical estrogen signaling pathways. Future work could benefit from comparing the 12Z cell line to primary cell models with better-maintained hormonal sensitivity.

Our study has a few limitations. It is unclear whether the presence of stromal cells contributes to the sensitivity of epithelial endometriotic cells to E2. Currently, there is no well-established endometriotic stromal cell line. However, future studies should consider testing E2 sensitivity in a co-culture of 12Zs and primary endometriotic stromal cells compared to each cell type individually. Another limitation of our study is that we obtained our 12Z cell line at passage 55. It is unknown how long the 12Z cell line can be passaged before it starts losing its original phenotype, but some changes have likely occurred: we detected nearly absent levels off ER⍺ in the 12Zs (Figure 4A,B), contrary to earlier reports of ER⍺ levels in the 12Zs [6,7]. The restricted E2 responsiveness observed here is a characteristic of the high-passage 12Z population evaluated in this study; whether it is an intrinsic property of all 12Z cells is unclear. We did not have access to earlier passages of the 12Zs, but it would be beneficial for others who do to study the effects of passaging on the retention of key traits of this particular cell line. In our dose–response experiments (Figure 1), we only included concentration-matched vehicle controls for the lower and upper doses of E2. For a more thorough understanding of vehicle effects, it would be insightful to have concentration-matched vehicle controls for every concentration of E2 used. Also, ERα Western blot quantification was normalized to α-SMA, which may vary with cellular phenotype, although equal total protein input was determined by BCA assay prior to electrophoresis. Therefore, the precise fold-change in ERα abundance between parental and ESR1-12Z cells may be further validated with an alternate reference protein. Additionally, the *ESR1*-12Z cell line was not accompanied by an empty-vector stable control line; therefore, differences between *ESR1*-12Zs and normal 12Zs could reflect effects of stable transfection/selection or passage differences in addition to *ESR1* upregulation. Lastly, RNA sequencing was performed on three pooled trials, resulting in one pooled sequencing library per condition. The RNA-sequencing results are therefore intended to be exploratory and hypothesis-generating.

## 5. Conclusions

In conclusion, while 12Z cells are a widely used in vitro model of endometriosis, our findings indicate a restricted and context-specific responsiveness to E2. This selective behavior, especially under physical confinement, warrants further investigation into whether and how physical context influences hormonal signaling. However, researchers should exercise caution when using this cell line to model estrogen-dependent processes and consider verifying hormone sensitivity in their specific experimental context.

## Data Availability

The data presented in this study are available from the corresponding author upon reasonable request.

## Supplementary Materials

The following supporting information can be downloaded at https://www.mdpi.com/article/10.3390/cells15201875/s1, Supplementary Material S1: Methods; Material S2: Functional assessment of alternative source of E2 on 12Zs; Material S3: Western Blots; Material S4: Gene expression analysis VE_12Zs vs. E2_12Zs; Material S5: Gene expression analysis NT_12Z vs. ESR1_12Z; Material S6: KEGG pathways.
