# Supplementary material for: Selective Estradiol Sensitivity in 12Z Human Endometriotic Epithelial Cell Line
Source: Cells. 2026 Oct 15;15(20):1875. doi: 10.3390/cells15201875 (PMC13703101; doi:10.3390/cells15201875)
Supplement: Supplementary file 1 [file cells-15-01875-s001.zip › S2. Functional assessment of alternative source of E2 on 12Zs.pdf]

SUPPLEMENTARY MATERIAL S2

Functional assessment of alternative source of E2 on 12Zs

We found that the 12Z cell line demonstrated a limited responsiveness to E2 treatment in proliferation and migration assays (Fig. 2) which prompted us to question the reliability of our source of E2. We obtained a new vial of 17β-estradiol (MP Biomedicals), denoted MPB E2. We treated 12Zs with 10 nM MPB E2 or the vehicle control (0.001% EtOH) for 48 hr and tested their functional response. MPB E2 had no effect on wound closure (Fig. S2A) but did significantly increase transmigration in a transwell assay (Fig. S2B). These trends matched our results using the original source of E2 (Sigma Aldrich, cat. #E8875-1G), as seen in Fig. 2B and Fig. 5A. Therefore, we found no reason to question the integrity of the E2 from Sigma Aldrich, whose Certificate of Analysis reports a purity greater than 98% by high-performance liquid chromatography.

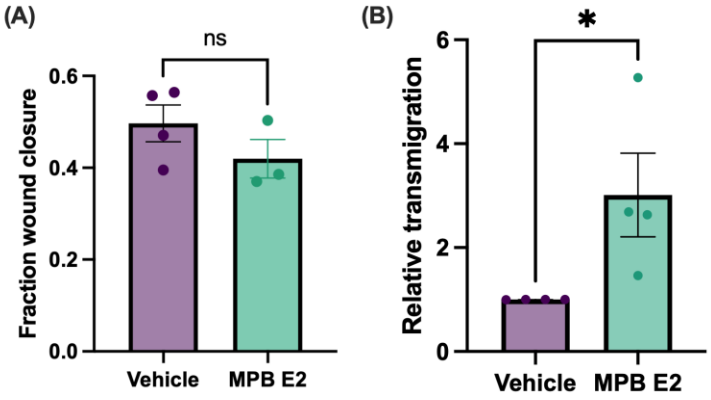

Figure S2. Functional assessment of alternative source of E2 on 12Zs. E2 purchased from MP Biomedicals (MPB E2) was applied to 12Zs and their functional response was determined. **A)** Fraction of wound closed is reported and **B)** transwell migration is reported relative to the vehicle control. Data points represent biological replicates, error bars represent standard error of the mean. *$P \leq 0.05$. MPB E2; MP Biomedicals estradiol.
