# Supplementary material for: Selective Estradiol Sensitivity in 12Z Human Endometriotic Epithelial Cell Line
Source: Cells. 2026 Oct 15;15(20):1875. doi: 10.3390/cells15201875 (PMC13703101; doi:10.3390/cells15201875)
Supplement: Supplementary file 1 [file cells-15-01875-s001.zip › S3. Western Blots.pdf]

**SUPPLEMENTARY MATERIAL S3**

**Western Blots**

*ESR1*-12Zs acquired from Dr. Fazleabas's lab (Michigan State University) were tested against 12Zs to verify the upregulation of ERα. We also tested the effect of E2 on ERα expression in both cell lines. Western blots were performed in triplicate (Fig. S3A). E2 had no effect on ERα expression in 12Zs (Fig. S3B,A) or *ESR1*-12Zs (Fig. S3B,B).

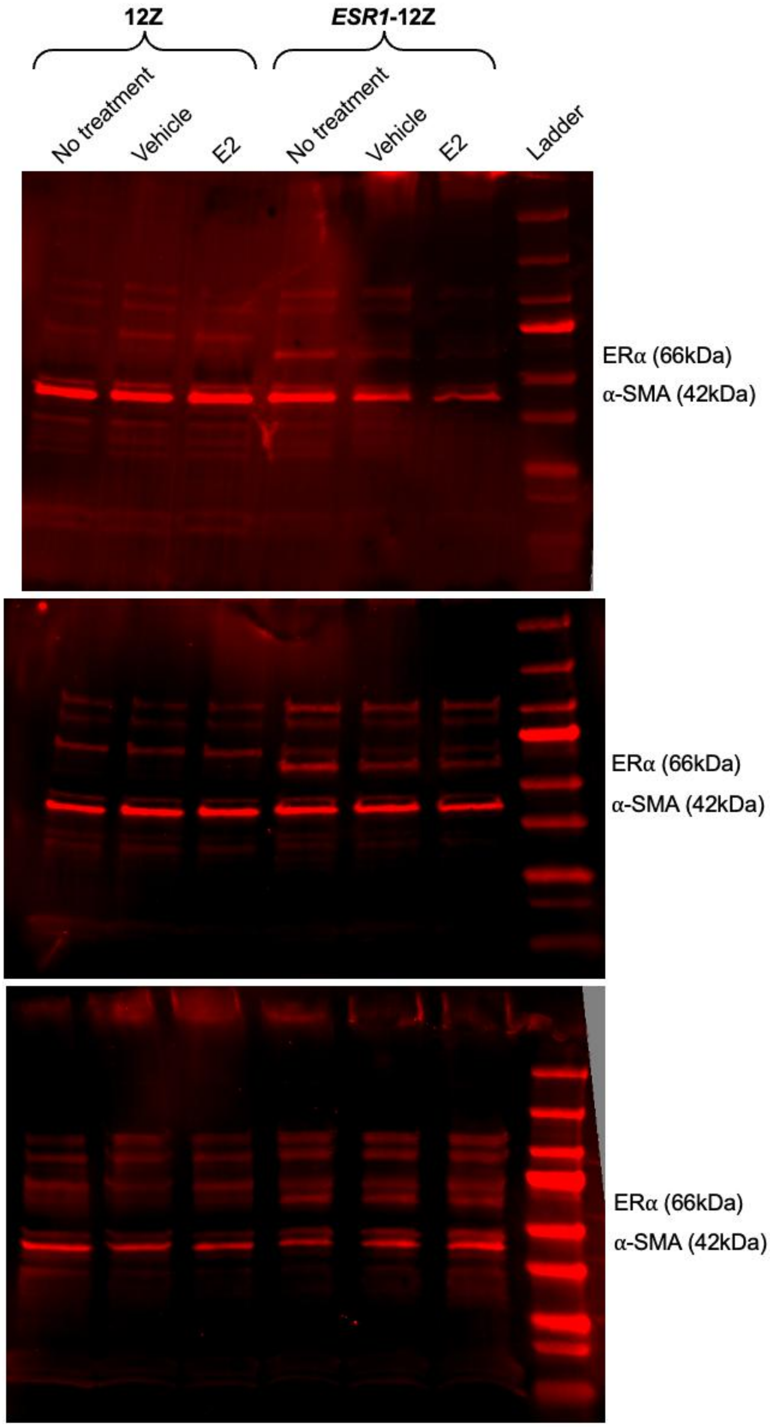


**Figure S3A. Western blots measuring upregulation of ERα in *ESR1*-12Zs.** Western blots were performed using α-SMA (42kDa) as the reference protein and the SeeBlue Plus2 prestained protein ladder. Quantification of ERα expression in *ESR1*-12Zs and 12Zs (no treatments) is shown in the main article in

Fig. 4B. Blots are from three independent trials ($n$ = 3). E2; estradiol, ERα; estrogen receptor-α, α-SMA; α-smooth muscle actin.

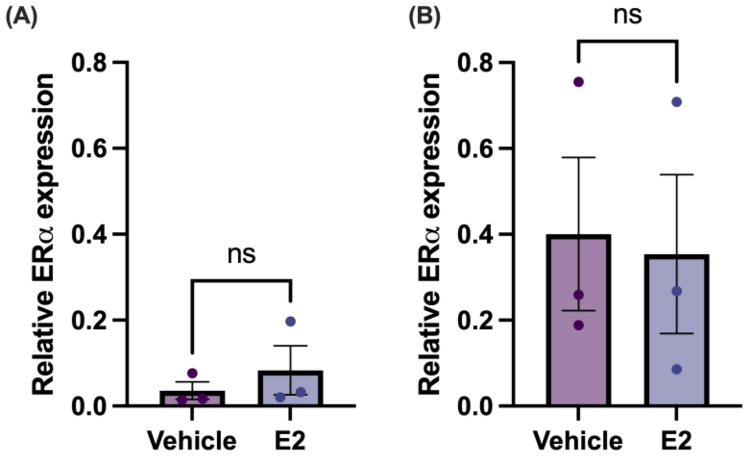


**Figure S3B. Effect of E2 on ERα expression in 12Zs and *ESR1*-12Zs.** Cells were treated with vehicle (0.001% EtOH) and 10nM E2 for 48 hr prior to lysis. ERα expression was quantified relative to α-SMA expression in **A)** 12Zs and **B)** *ESR1*-12Zs. Data points represent biological replicates ($n$ = 3), error bars represent standard error of the mean. ns; not significant, E2; estradiol.
